# Comprehensive Bioinformatic Assessments of the Variability of Neisseria gonorrhoeae Vaccine Candidates

**DOI:** 10.1128/mSphere.00977-20

**Published:** 2021-02-03

**Authors:** Benjamin I. Baarda, Ryszard A. Zielke, Alaina K. Holm, Aleksandra E. Sikora

**Affiliations:** a Department of Pharmaceutical Sciences, College of Pharmacy, Oregon State University, Corvallis, Oregon, USA; b Vaccine and Gene Therapy Institute, Oregon Health and Science University, Beaverton, Oregon, USA; U.S. Food and Drug Administration

**Keywords:** gonorrhea, protein subunit vaccine, antigen variability, PubMLST, bioinformatics, crystal structure, phylogenetics, *Neisseria gonorrhoeae*, antigenic variation, phylogenetic analysis, subunit, vaccine

## Abstract

A protective vaccine is the only viable way to stop the spread of gonorrhea in the face of rising antibiotic resistance. However, the notorious phase and antigenic variation of Neisseria gonorrhoeae surface proteins remains one of the challenges in vaccine development. To facilitate vaccine advancement efforts, we carried out comprehensive bioinformatic analyses of sequence variation by comparing 34 gonorrhea antigen candidates among >5,000 clinical N. gonorrhoeae isolates deposited in the *Neisseria* PubMLST database. Eight protein antigens showed exceptional conservation by having a single allele variant distributed in >80% of isolates. An additional 18 vaccine candidates were represented by ≤3 alleles in >50% of N. gonorrhoeae isolates globally. Phylogenetic analyses highlighted closely related antigen variants and additionally showed that AniA and FetB were the closest between N. gonorrhoeae and N. meningitidis. Up to 44% of N. meningitidis alleles for both antigens have premature stop codons, suggesting differential expression. Mapping polymorphisms to the available three-dimensional structures of 12 antigens revealed low-frequency surface polymorphisms. PorB and TbpB possessed numerous high-prevalence polymorphic sites. While TbpA was also highly variable, conserved loops were nonetheless identified. A high degree of sequence conservation, the distribution of a single antigen variant among N. gonorrhoeae strains globally, or low-frequency sequence polymorphisms in surface loops make ACP, AniA, BamA, BamE, MtrE, NspA, NGO0778, NGO1251, NGO1985, OpcA, PldA, Slam2, and ZnuD promising candidates for a gonorrhea vaccine. Finally, the commonly used N. gonorrhoeae FA1090 strain emerges as a vaccine prototype, as it carries antigen sequence types identical to the most broadly distributed antigen variants.

**IMPORTANCE** Neisseria gonorrhoeae, the Gram-negative bacterium responsible for the sexually transmitted infection gonorrhea, is categorized as a high-priority pathogen for research and development efforts. N. gonorrhoeae’s “superbug” status, its high morbidity, and the serious health impact associated with gonorrhea highlight the importance of vaccine development. One of the longstanding barriers to developing an effective vaccine against N. gonorrhoeae is the remarkable variability of surface-exposed antigens. In this report, we addressed this roadblock by applying extensive bioinformatic analyses to 34 gonorrhea antigen candidates among >5,000 clinical N. gonorrhoeae isolates. Our studies are important, as they reveal promising, conserved gonorrhea vaccine candidates and aid structural vaccinology. Moreover, these approaches are broadly applicable to other infectious diseases where surface antigen variability impedes successful vaccine design.

## Abstract (precis)

Neisseria gonorrhoeae, the Gram-negative bacterium responsible for the sexually transmitted infection gonorrhea, is categorized as a high-priority pathogen for research and development efforts. N. gonorrhoeae’s “superbug” status, its high morbidity, and the serious health impact associated with gonorrhea highlight the importance of vaccine development. One of the longstanding barriers to developing an effective vaccine against N. gonorrhoeae is the remarkable variability of surface-exposed antigens.

## INTRODUCTION

Development of vaccines against many infectious diseases and cancers has been hampered by immune system evasion strategies, including variability of surface antigens (1–3). The pathogenic *Neisseria* species, Neisseria gonorrhoeae and Neisseria meningitidis, are notorious for their ability to alter their surface proteome. Both N. gonorrhoeae and N. meningitidis have three important surface structures capable of phase and antigenic variation (2), namely, lipooligosaccharide (LOS) glycan (4), opacity proteins (5), and type IV pilus (6), as well as 50 to 100 genes that undergo solely phase variation (2, 7). The success of vaccines against N. meningitidis serogroup B infections demonstrated that creative vaccine development strategies are necessary to circumvent the difficulties posed by antigen variability. Through reverse and rational vaccinology approaches, two meningococcal subunit vaccines, Bexsero (GSK; also known as 4CMenB) (8, 9) and Trumenba (Pfizer) (8, 10, 11), were developed and entered the market in 2014. Bioinformatic assessment of factor H binding protein conservation revealed its two immunologically distinct subfamilies, which prompted the inclusion of a representative of each subfamily in the final Trumenba vaccine (10–12). These successes suggest that a similar approach could benefit gonorrhea vaccine research, which has been focusing on a handful of antigens for years (13).

N. gonorrhoeae is classified as one of the five “urgent” antibiotic-resistant threats in the United States by the Centers for Disease Control and Prevention (CDC) (14). Until recently, the sole treatment regimen approved by the CDC was azithromycin combined with ceftriaxone (15, 16). However, this recommendation was changed to a single dose of ceftriaxone based on the increased occurrence of azithromycin resistance, the low incidence of ceftriaxone resistance, and antimicrobial stewardship concerns (17). A vaccine appears as the only sustainable line of defense against the detrimental health effects caused by the gonococcus, which include urethritis, cervicitis, proctitis, neonatal conjunctivitis and blindness, mucosal infection of the rectum and pharynx, and the facilitation of HIV transmission (18–21). The common hallmarks of urethral infection in men are purulent exudate and painful urination, while women may experience mild and nonspecific symptoms as well as dysuria and vaginal discharge (22, 23). However, many men and most women are asymptomatic (24, 25). If left untreated, gonorrhea can result in complications, including epididymitis, endometritis, pelvic inflammatory disease, and ectopic pregnancy (26, 27). In many countries, gonorrhea case rates are increasing and disparities in the overall disease rate and burden persist among sexual, gender, racial, and ethnic minorities as well as resource-limited populations (14, 28, 29). The CDC, its European counterpart, the ECDC, and the World Health Organization (WHO) have all emphasized the dire threat posed by drug-resistant gonorrhea (26, 27, 30). Vaccine development efforts must be expanded and accelerated to slow the spread of this disease (26).

Ever more powerful and useful genomics, proteomics, and bioinformatics tools are instrumental for broadening the repertoire of gonorrhea vaccine candidates and facilitating vaccine development. For instance, quantitative proteomic and immunoproteomic studies identified over 20 new gonorrhea vaccine candidates. These proteins are present at similar levels in N. gonorrhoeae cell envelopes of 19 diverse isolates (31, 32), including the 2016 WHO reference strains, and in native outer membrane vesicles (OMVs) of four commonly used laboratory strains (32). Further, high-throughput proteomics revealed their expression profiles under conditions relevant to host infection, including iron starvation, exposure to human serum, or anaerobiosis (33). These studies combined with data from omics profiling of N. gonorrhoeae biofilms and during host infection provide invaluable insights about expression of all gonorrhea antigens currently being pursued in preclinical studies (13, 34–39). However, information about their antigenic variability globally, which is critically needed to identify conserved antigens with potential to develop broadly protective immunity against the highly variable N. gonorrhoeae, remains scarce. To address this gap, in this report we assessed the conservation of 34 candidate vaccine antigens discovered through different traditional (13) and proteomics-based reverse vaccinology efforts (31–33). We examined sequence variation and phylogenetic relationships among alleles for a single protein using sequence data from >5,000 N. gonorrhoeae isolates in the publicly available *Neisseria* multilocus sequence typing database (*Neisseria* PubMLST; https://pubmlst.org/organisms/neisseria-spp, sited at the University of Oxford) (40). This database contains sequence data from over 60,000 isolates of pathogenic and commensal *Neisseria* species collected worldwide, providing a rich resource for antigen diversity mining. We additionally mapped amino acid polymorphic sites to available antigen crystal structures. This approach provides a visual representation of protein conservation and illuminates conserved surface loops that could be incorporated into a vaccine delivery platform, thereby facilitating structural vaccinology. Polymorphic amino acids were mapped based on their prevalence in the population, rather than on the raw number of sequences available, to give more relevant insights about allele distribution. Finally, we also performed comparative analyses between each gonococcal protein antigen and its meningococcal homolog, to determine the level of conservation between the two species and to identify candidates for inclusion in a cross-protective vaccine against both N. gonorrhoeae and N. meningitidis.

The approaches we describe for N. gonorrhoeae are broadly applicable, as the PubMLST family of databases is home to sequence data from over 100 species and genera, including prokaryotic and eukaryotic pathogens (40, 41). New isolates are added to the database frequently, providing an up-to-date understanding of global protein variation.

## RESULTS AND DISCUSSION

### Antigen selection and workflow for bioinformatics mining.

**FIG 1:**
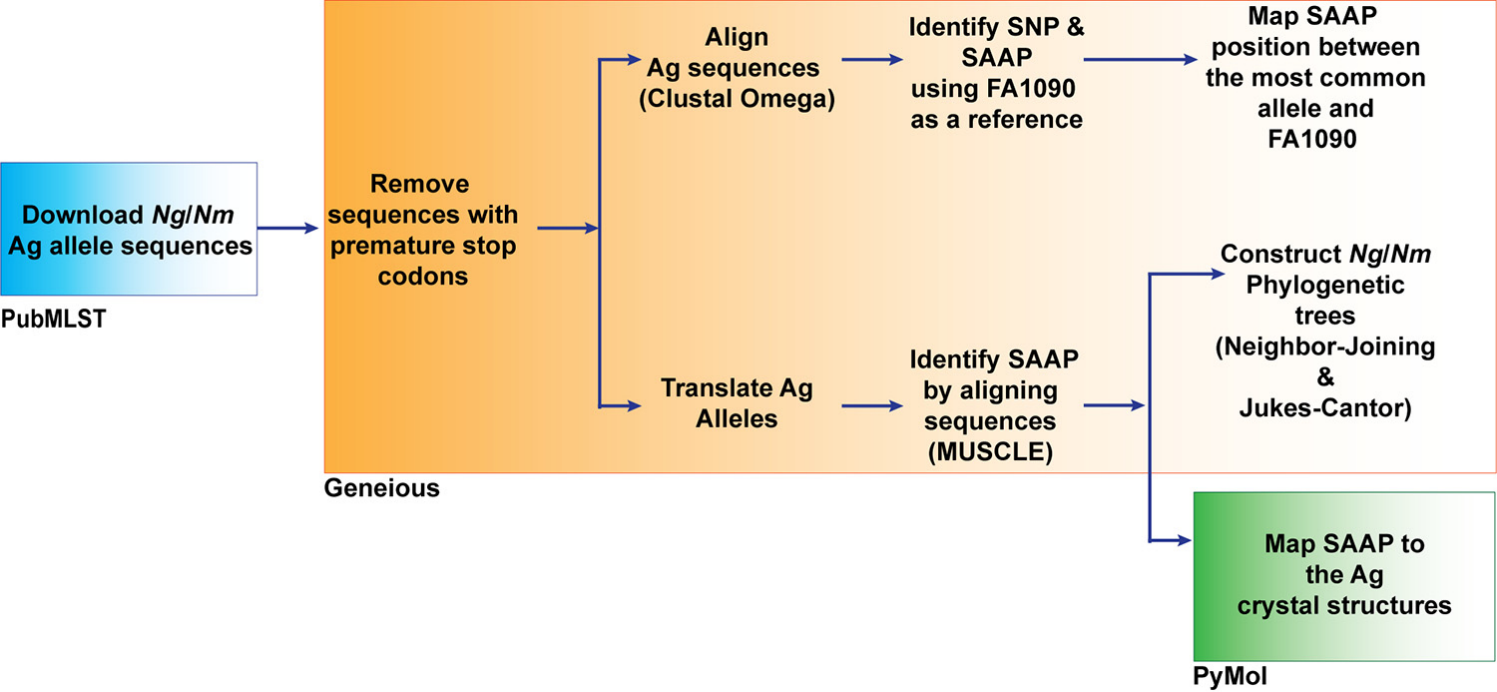
Workflow for N. gonorrhoeae antigen variability mining. The N. gonorrhoeae and N. meningitidis (*Ng*/*Nm*) antigen (Ag) nucleotide sequences were downloaded from the PubMLST database (blue box). Geneious Prime 2020.1.2 software was subsequently used to perform analyses described below and outlined in the orange box. Sequences containing premature stop codons were eliminated. Nucleotide sequences were aligned using the Clustal Omega algorithm. To identify SNPs and SAAPs from the deduced amino acid sequence compared to the FA1090 allele sequence as a prototype, the Annotate & Predict tool was applied. In parallel, nucleotide sequences were translated to their respective amino acid sequences and aligned using the MUSCLE algorithm, followed by generation of neighbor-joining phylogenetic trees with the Jukes-Cantor genetic distance model. For proteins with available structural data, the proportion of isolates associated with each SAAP compared to the most common amino acid was calculated. Polymorphisms present in ≥1% of isolates were mapped to the protein three-dimensional structure using PyMOL (green box). SNP, single nucleotide polymorphism; SAAP, single amino acid polymorphism.

We selected the 34 gonorrhea protein antigens that were discovered through traditional approaches (13) and proteome-based reverse vaccinology studies (31–33). Excluded were the highly polymorphic opacity-associated proteins, candidates identified experimentally to be periplasmic, MetQ and MlaA (reported in references 38 and 42), and the small lipoprotein NGO2054 (33, 43) that has inconsistent annotations in PubMLST (e.g., annotated independently, as part of NGO2055, or lacks annotation). For comprehensive bioinformatics mining of N. gonorrhoeae antigens, we have developed the workflow presented in Fig. 1. The antigen sequences were downloaded according to the detailed instructions we previously described (41). Eight of the 34 antigens, including LbpA, PilQ, IgA2 protease, CsgG, MtrE, OpcA, TbpB, and ZnuD, contained various numbers of alleles disrupted by premature termination codon (see Table S1 in the supplemental material). The highest numbers of these alleles, 31, 18, and 16, were identified for LbpA, PilQ, and IgA2 protease, respectively. The presence of a premature stop codon may suggest phase variation events and most commonly results in loss of protein expression, a nonfunctional truncated protein, or, rarely, a protein with altered function (44). Therefore, alleles containing premature stop codons (89 in total) were excluded from further analysis. The N. gonorrhoeae strain FA1090 was used as a reference to identify the single nucleotide polymorphisms (SNPs) and single amino acid polymorphisms (SAAPs) in the obtained antigen sequences (Data Set S1). For each antigen, we mapped SAAPs identified in the most frequent antigen variant to the corresponding allele in N. gonorrhoeae FA1090 (Table 1). Finally, based on the prevalence data available from PubMLST, the frequencies of each SAAP were calculated, and the most common polymorphisms (found in ≥1% of the global population) were mapped to available protein crystal structures, either from N. gonorrhoeae or the closely related N. meningitidis (see Fig. 6 and 7).

**TABLE 1:** Summary of antigen polymorphism analyses^*a*^

Antigen (length range; mean mol wt)	No. of alleles	No. of SNPs	No. of unique amino acid sequences	No. of SAAPs	Position(s) of polymorphism(s) between FA1090 and most common allele (residue no. FA1090_aa_ » allele_aa_)^*b*^
ACP (123–124 aa; 13.3 kDa)	22	41	14	16	20 A»−; 25 N»D
AniA (386–402 aa; 40.9 kDa)	101	233	52	59	323 N»S
BamA (791–793 aa; 88 kDa)	171	130	78	65	553 R»K; 554 K»Q; 585 S»L
BamE (125 aa; 13.9 kDa)	8	38	6	13	NA (FA1090 allele is most common)
CsgG (NGO0834) (220–224 aa; 23.7 kDa)	24^*c*^	44	19	25	119 V»I
FetB (NGO2092) (287–337 aa; 34.1 kDa)	163	77	71	35	6 A»T; 107 D»E; 193 G»−; 194 K»−
IgA1 protease (1,533–1,595 aa; 173.6 kDa)	297	815	248	324	142 S»G; 467 S»A; 468 N»D; 469 Q»K; 491 P»S; 492 D»N; 497 N»D; 567 N»D; 609 Q»V; 610 T»A; 616 I»V; 617 L»F; 620 S»Y; 827 Q»K
IgA2 protease (1,461–1,476 aa; 160.4 kDa)	174^*c*^	227^*c*^	116	106^*c*^	1271 G»E
LprI (339 aa; 36.8 kDa)	35	37	25	21	NA (no amino acid polymorphic sites between most common allele and FA1090 allele)
LptD (797–805 aa; 87.5 kDa)	183^*d*^	500^*d*^	85	196^*d*^	51 S»F; 97 E»Q; 101 K»Q; 446 H»Q
MafA (NGO1067) (313–321 aa; 34.6 kDa)	89	228	30	122	283 Q»R
MtrE (467–483 aa; 50.5 kDa)	64^*c*^	148^*c*^	36	40^*c*^	47 I»V; 454 V»A
NGO1985 (203 aa; 21.7 kDa)	14	37	10	16	NA (no amino acid polymorphic sites between most common allele and FA1090 allele)
NGO0425 (208–209 aa; 23.149 kDa	24	47	17	25	NA (no amino acid polymorphic sites between most common allele and FA1090 allele)
NGO0778 (144 aa; 16.1 kDa)	13	12	10	9	NA (FA1090 allele is most common)
NGO1559 (223–225 aa; 23.5 kDa)	32	30	23	22	7 F»S
NGO1251 (191–192 aa; 21.8 kDa)	18	29	8	14	NA (no amino acid polymorphic sites between most common allele and FA1090 allele)
NGO1344 (700–742 aa; 77.2 kDa)	119	266	50	150	NA (no amino acid polymorphic sites between most common allele and FA1090 allele)
NgMIP (NGO1225) (269–278 aa; 28.9 kDa)	36	51	21	21	49 S»G; 160 E»K
NspA (NGO0233) (173–175 aa; 18.4 kDa)	29*^c,d^*	22*^c,d^*	20	14	153 V»I
LbpA (881–952 aa; 105.7 kDa)	122	175	62	83	Internal stop codon for FA1090; SNP and nsSNP analysis done against most common allele
LolB (NGO0439) (193 aa; 21.2 kDa)	25	33	20	21	74 S»G
OmpU (NGO1688) (485–491 aa; 56.5 kDa)	165	200	123	119	37 C»R; 213 V»I; 270 V»A
OpcA (NGO0868) (262–264 aa; 28.9 kDa)	54^*c*^	27	30	18	100 E»K,116 I»L, 192 V»A
PilN (NGO0097) (199 aa; 22.2 kDa)	38	29	23	18	92 E»G
PldA (373–376 aa; 42 kDa)	27	37	16	16	NA (FA1090 allele is most common)
PorB (326–357 aa; 37.1 kDa)	1,262	507	1,229	269	18 M»T; 40 T»R; 41 D»E; 45 S»I; 46 K»G; 48 E»G; 54 A»S; 75 V»I; 89 T»S; 98 V»I; 120 G»K; 121 A»N; 187 Q»R; 189 S»N; 208 K»−; 209 I»−; 210 E»−; 211 Y»M; 212 D»E; 214 Q»Y; 215 T»A; 217 S»N; 242 V»A; 272 A»V; 295 S»D;
Slam2 (505–511 aa; 55.6 kDa)	53	483	41	181	NA (no amino acid polymorphic sites between most common allele and FA1090 allele)
SliC (122–126 aa; 13.5 kDa)	12	22	8	11	NA (FA1090 allele is most common)
TamA (610–616 aa; 67.7 kDa)	140	174	72	69	361 R»Q
TbpA (905–936 aa; 102.1 kDa)	638^*c*^	592^*c*^	573	217^*c*^	110 A»S; 247 V»A; 257 Y»H; 262 E»A; 265 K»E; 267 E»G; 268 G»S; 271 K»T; 274 A»T; 275 −»R; 276 −»P; 281 D»A; 356 E»A; 358 K»Q; 359 −»K; 360 K»Q; 361 Y»A; 364 I»L; 367 Y»N; 373 G»N; 374 R»H; 377 S»G; 380 I»F; 382 N»S; 386 G»N; 388 E»P; 421 T»A; 463 S»F; 509 D»N; 519 H»S; 530 P»A; 539 −»P; 540 −»D; 555 G»R; 561 G»R; 630 T»A; 665 G»D; 699 N»D; 712 A»V; 720 Q»G; 836 D»N; 887 A»G;
TbpB (691–703 aa; 74.7 kDa)	320^*c*^	1792^*c*^	311	219^*c*^	40 P»A; 74 P»R; 75 P»M; 76 S»A; 78 P»−; 79 K»−; 83 I»V; 84 R»K; 86 S»N; 88 G»S; 94 G»D; 100 N»E; 106 N»S; 112 S»T; 115 G»D; 116 E»G; 117 A»E; 118 P»T; 125 Q»−; 126 G»Q; 134 D»G; 149 Q»H; 151 G»R; 152 N»T; 153 T»K; 154 I»P; 155 K»E; 156 K»−; 157 D»−; 158 D»−; 159 S»T; 160 S»I; 161 S»D; 162 K»G; 163 I»K; 164 I»V; 165 E»T; 166 A»V; 168 N»S; 190 S»K; 192 E»M; 204 Q»K; 214 S»N; 216 F»S; 217 T»A; 219 S»P; 235 R»K; 238 E»D; 256 N»D; 258 E»G; 300 M»I; 301 V»A; 306 E»K; 307 N»A; 308 S»N; 309 K»E; 310 S»T; 312 Q»E; 330 Q»K; 341 N»D; 343 N»G; 373 N»G; 375 A»T; 379 S»P; 381 G»E; 382 N»−; 383 S»N; 384 N»K; 400 E»K; 424 S»P; 436 K»E; 439 G»K; 441 D»A; 443 T»I; 445 T»E; 449 M»T; 458 K»Q; 459 A»T; 460 Q»G; 461 T»M; 462 G»A; 463 A»T; 464 G»N; 466 M»V; 469 A»V; 471 D»N; 472 A»T; 475 V»G; 476 N»T; 477 G»S; 479 Q»K; 480 A»T; 481 G»−; 482 T»K; 483 K»T; 484 T»H; 488 E»Q; 505 E»K; 508 N»E; 510 V»A; 513 T»A; 514 V»G; 515 R»E; 516 N»S; 519 Q»R; 520 A»T; 522 A»V; 523 R»Q; 546 E»D; 547 Q»G; 548 K»N; 561 A»I; 568 G»R; 569 N»E; 572 D»N; 574 Q»E; 575 S»N; 611 D»E; 612 D»G; 623 N»D; 624 D»G; 632 S»N; 637 Y»H; 643 N»E; 645 E»K; 670 T»A; 671 K»E; 673 A»−; 674 Q»−; 675 E»−; 676 N»−; 679 A»T; 688 V»G; 700 Q»E
ZnuD, TdfJ (NGO1205) (763–764 aa; 85.7 kDa)	161^*c*^	93^*c*^	52	47	658 A»D

a Antigens are listed in alphabetical order. aa, amino acids.

b Residue number includes gaps only if present in one of the two sequences. Dashes indicate a single amino acid deletion (gap). NA, not applicable; nsSNP, nonsynonymous single-nucleotide polymorphisms.

c Includes only those alleles without an annotated premature stop codon.

d Excludes allele annotated as atypical.

10.1128/mSphere.00977-20.1 TABLE S1 Antigens with altered alleles. Alleles containing an internal stop codon were eliminated from further analysis. Download Table S1, PDF file, 0.02 MB. Copyright © 2021 Baarda et al. 2021 Baarda et al. This content is distributed under the terms of the Creative Commons Attribution 4.0 International license.

10.1128/mSphere.00977-20.10 DATA SET S1 SNP/SAAP analyses comparing all alleles to the most common allele (LbpA) or to the FA1090 allele (all others). Download Data Set S1, XLSX file, 0.5 MB. Copyright © 2021 Baarda et al. 2021 Baarda et al. This content is distributed under the terms of the Creative Commons Attribution 4.0 International license.

### NGO0778, SliC, PldA, NGO1985, BamE, ACP, Slam2, and NGO1251 are highly conserved gonorrhea vaccine candidates.

**FIG 2:**
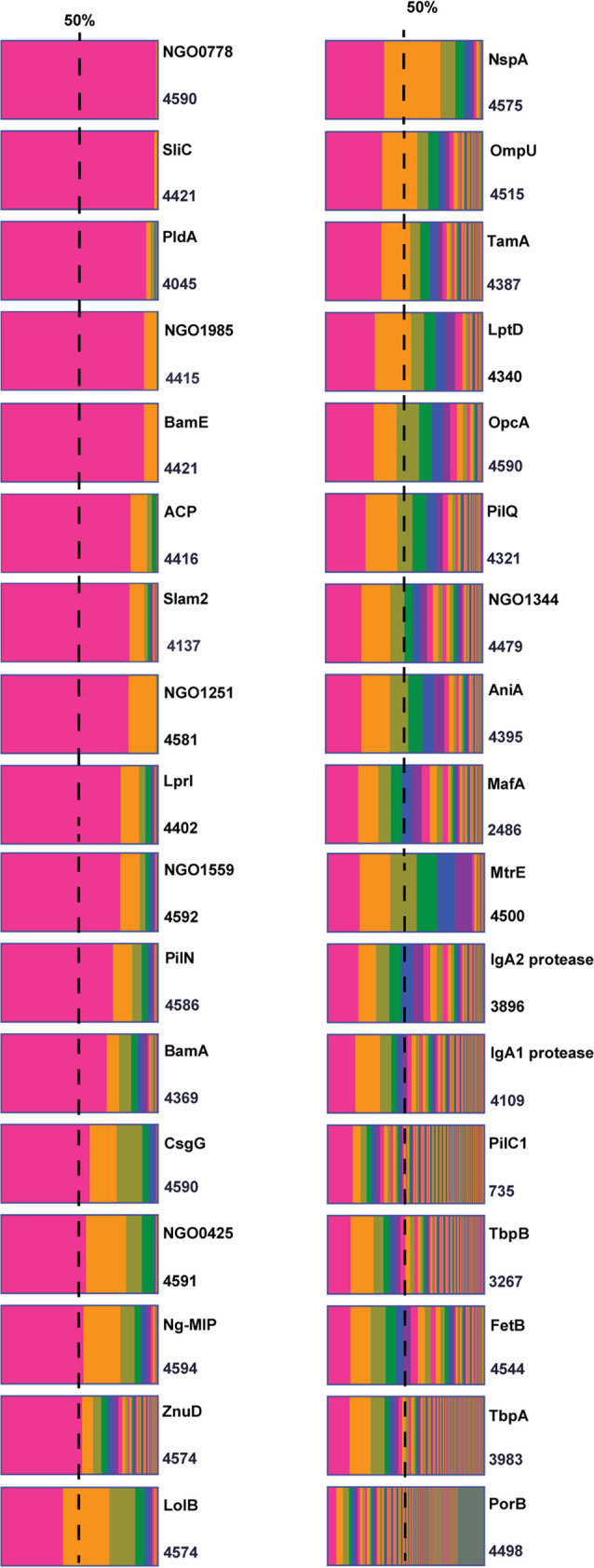
Distribution of each antigen nucleotide sequence variant in the global N. gonorrhoeae population. The percentages of N. gonorrhoeae isolates associated with each antigen allele were generated using GraphPad Prism 9. The total number of isolates with data for the antigen is indicated at the bottom right of each graph. The dotted line represents 50% of N. gonorrhoeae isolates. Antigens are arranged based on the prevalence of the most common allele in the N. gonorrhoeae global population.

To illustrate the distribution of each antigen nucleotide sequence variants in the global N. gonorrhoeae population, we graphed the percentage of isolates associated with each antigen allele (Fig. 2). Remarkably, over 90% of isolates carry an identical allele of NGO0778, SliC, PldA, NGO1985, and BamE. Further, between 80% and 90% of N. gonorrhoeae strains have a sole nucleotide sequence for acyl carrier protein (ACP), Slam2, and NGO1251. For 18 additional antigens—LprI, NGO1559, PilN, BamA, CsgG, NGO0425, Ng-MIP, ZnuD, LolB, NspA, OmpU, TamA, LptD, OpcA, PilQ, NGO1344, AniA, and MtrE—three or fewer alleles encompass >50% of N. gonorrhoeae isolates worldwide.

As expected, at the protein level, less unique SAAPs are present in the population for each antigen due to the presence of synonymous mutations. Thus, fewer protein variants would need to be included in a vaccine than the number of alleles would suggest (Table 1; Data Set S1). The most highly conserved antigen was BamE, with eight alleles and six distinctive amino acid sequences (Table 1; Data Set S1). Eight different amino acid sequences were present for SliC and NGO1251, whereas 10, 10, and 14 distinct amino acid sequence variants occurred for NGO1985, NGO0778, and ACP, respectively. The least conserved antigen, with 1,262 alleles and 1,229 unique amino acid sequences, was PorB. The dramatic difference in conservation between antigens illustrates the importance of this approach to evaluate the variability of candidate antigens before their testing in preclinical studies and inclusion in a vaccine. Our results showed that the FA1090 amino acid sequence is the most prevalent globally in 10 of the 34 antigens analyzed and diverges from the most common amino acid sequence by one or two polymorphisms in an additional 13 antigens (Table 1). The sole exception is LbpA, which has a premature stop codon in the FA1090 allele. Therefore, for this locus we used as the reference sequence the most common LbpA allele 16, which is carried, for instance, by the 2016 WHO reference strains WHO-M, WHO-P, WHO-U, and WHO-Y (45).

Cumulatively, these results suggest that the eight well-conserved proteins with >80% global homogeneity have the potential to protect against a wide range of N. gonorrhoeae strains if they are strong immunogens that are expressed on the bacterial cell surface during host infection. A combination of two or more conserved antigens could increase the vaccine coverage and effectiveness in comparison to a single-component vaccine. An example of this strategy is the N. meningitidis serogroup B vaccine Trumenba (Pfizer), which includes two representative factor H binding protein (fHbp) variants to provide broader coverage (10, 11). Finally, the antigen conservation data further validate the choice of FA1090 as a vaccine antigen prototype and a challenge strain in immunization/challenge experiments (32, 33, 36, 38, 39, 41, 46).

### Phylogenetic analyses of gonorrhea vaccine candidates reveal evolutionarily distinct groups.

**FIG 3:**
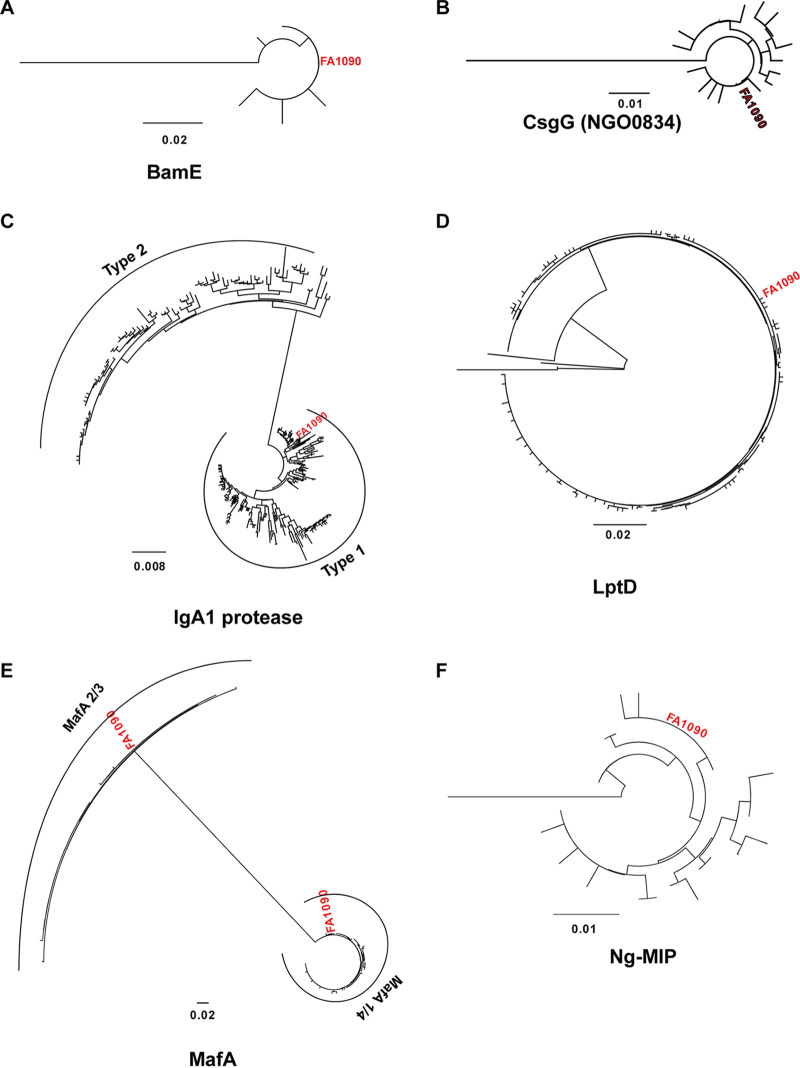
Phylogenetic relationships of BamE, CsgG, IgA1 protease, LptD, MafA, and Ng-MIP among N. gonorrhoeae isolates. Neighbor-joining phylogenetic trees of antigens (as indicated below each tree) from N. gonorrhoeae were constructed in Geneious using the Jukes-Cantor genetic distance model. The FA1090 allele is designated in red text for each antigen. Subfamilies, when established, are indicated with curved lines. Antigens are organized in alphabetical order: BamE (A), CsgG (B), IgA1 protease (C), LptD (D), MafA (E), Ng-MIP (F).

**FIG 4:**
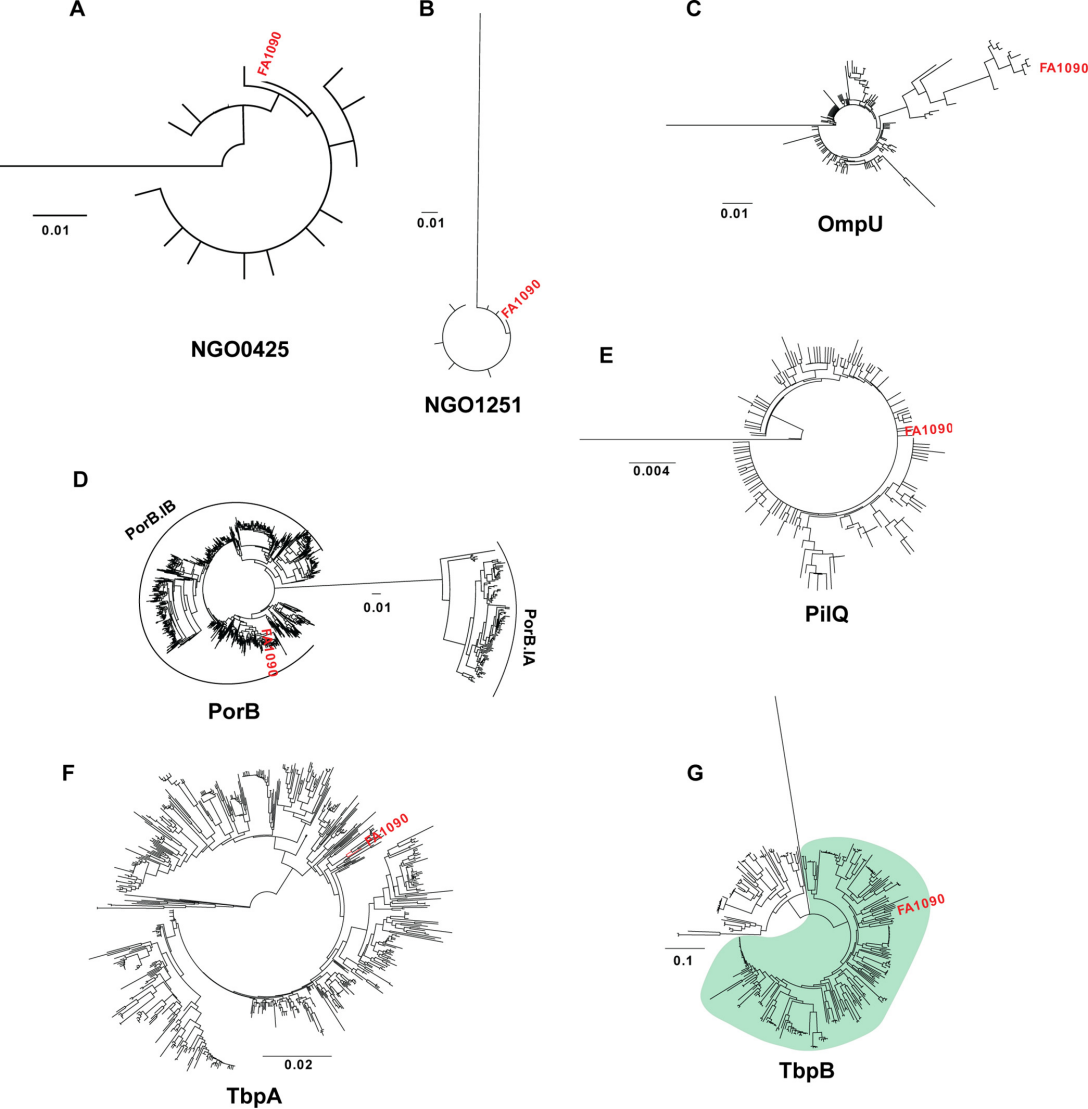
Phylogenetic relationships of NGO425, NGO1251, OmpU, PorB, PilQ, TbpA, and TbpB among N. gonorrhoeae isolates. Phylogenetic trees of antigens were constructed in Geneious. The FA1090 allele for each antigen is designated in red text. Subfamilies, when established, are indicated with curved lines. The green-shaded region of TbpB phylogeny indicates the TbpB_E_ subfamily. Antigens are listed in alphabetical order: NGO0425 (A), NGO1251 (B), OmpU (C), PorB (D), PilQ (E), TbpA (F), TbpB (G).

We constructed phylogenetic trees to determine the evolutionary relationship between alleles and to assess distinct N. gonorrhoeae antigen families (Fig. 3 and 4; Fig. S1 to S3). The results of our phylogenetic analyses reflect and expand upon our prior observations (Table 1 and Fig. 2) by illustrating the number of alleles and how closely related they are on the amino acid level. The majority of alleles for each antigen were closely related, with notable exceptions. Although it is the most highly conserved of the antigens examined, BamE has a single allele that is divergent from the rest and forms an outgroup (Fig. 3A). Similar single-allele outgroups are observed for CsgG (Fig. 3B), LptD (Fig. 3D), Ng-MIP (Fig. 3F), NGO0425 (Fig. 4A), NGO1251 (Fig. 4B), OmpU (Fig. 4C), PilQ (Fig. 4E), and TbpB (Fig. 4G). These outgroups represent a minimal proportion of N. gonorrhoeae isolates for each antigen. NGO0425 has the outgroup that encompasses the most isolates, at 13 (0.2% of the isolates with data for this locus).

10.1128/mSphere.00977-20.2 FIG S1 Phylogenetic relationships of vaccine candidates among N. gonorrhoeae isolates for antigens placed in alphabetical order from A to L. Neighbor-joining phylogenetic trees of N. gonorrhoeae antigens (as indicated below each tree and listed in alphabetical order from A to L) that were not included in the main text. The FA1090 allele is designated in red. Download FIG S1, PDF file, 0.1 MB. Copyright © 2021 Baarda et al. 2021 Baarda et al. This content is distributed under the terms of the Creative Commons Attribution 4.0 International license.

10.1128/mSphere.00977-20.3 FIG S2 Phylogenetic relationships of vaccine candidates among N. gonorrhoeae isolates organized in alphabetical order from M to O. Phylogenetic trees of N. gonorrhoeae antigens (in alphabetical order from M to O) that were not described in the main text. The FA1090 allele is designated in red text for each antigen. Download FIG S2, PDF file, 0.1 MB. Copyright © 2021 Baarda et al. 2021 Baarda et al. This content is distributed under the terms of the Creative Commons Attribution 4.0 International license.

10.1128/mSphere.00977-20.4 FIG S3 Phylogenetic relationships of vaccine candidates among N. gonorrhoeae isolates arranged in alphabetical order from P to Z. Neighbor-joining phylogenetic trees of N. gonorrhoeae antigens (listed alphabetically from P to Z) that were not mentioned in the main text of the article. The FA1090 allele is designated in red text for each antigen. Download FIG S3, PDF file, 0.2 MB. Copyright © 2021 Baarda et al. 2021 Baarda et al. This content is distributed under the terms of the Creative Commons Attribution 4.0 International license.

Our results are also consistent with the separation of several antigens into multiple subfamilies. We observed distinct subfamilies for IgA1 protease (Fig. 3C), MafA (Fig. 3E), PorB (Fig. 4D), and TbpB (Fig. 4G), discussed below. In our analysis, IgA1 protease clusters into two evolutionarily distinct groups, which correlate with the established classification into type 1 and type 2 IgA1 proteases (Fig. 3C) (47). Although both subfamilies function as autotransporter serine proteases that cleave the hinge region of the IgA1 heavy chain (48–52), they recognize different cleavage sites. Type 1 cleaves a proline-serine bond, and type 2 cleaves a proline-threonine bond immediately upstream of the type 1 recognition site (47). Further, the two variants have different secondary substrates (53–55), which may contribute to gonococcal virulence in ways that are not yet clear. Due to the evolutionary distance between the two protease types, a representative of each type will likely need to be considered for a vaccine, as N. gonorrhoeae isolates express one or the other but not both (47, 56).

MafA is an adhesin in the multiple adhesin family (*maf*) which binds to glycolipid cell receptors and mediates cell attachment (57). Up to five *maf* variants are encoded on horizontally acquired genomic islands in N. gonorrhoeae genomes (58). FA1090 possesses four copies of MafA: two pairs of identical loci, designated MafA 1/4 (NGO1067 and NGO1972) and MafA 2/3 (NGO1393 and NGO1584). Both pairs are returned as a single locus when the PubMLST database is queried. Our results show that the two MafA subtypes are evolutionarily distinct, while the alleles associated with each subtype are closely related (Fig. 3E). Therefore, a vaccine formulated with MafA should incorporate both subfamilies to account for isolates that may have one or the other.

The porin PorB (also known as protein I) is one of the most abundant proteins in the gonococcal cell envelope and acts primarily as a voltage-gated channel to facilitate ion exchange with the environment (59). N. gonorrhoeae isolates possess one copy of PorB in one of two allelic forms, PorB.IA or PorB.IB, which share only ∼70% nucleotide sequence identity (60). The two protein forms are associated with different disease states: PorB.IA isolates are generally associated with disseminated infections, while PorB.IB-expressing strains usually cause localized urogenital infections (61, 62). PorB subtypes also contribute to fitness in the host in different ways (63–66). Consistent with the presence of two PorB subtypes in the N. gonorrhoeae population, this phylogenetic analysis split PorB alleles into two distinct clusters that correlate with PorB.IA (Fig. 4D, small cluster) and PorB.IB (Fig. 4D, large cluster). Although PorB.IA accounts for <10% of PorB sequences worldwide (426 of 4,498 isolates with PorB sequence data), representatives from both groups could be included in a vaccine, due to the difference in secondary function and extensive sequence diversity between the subtypes, as well as the dangerous disease phenotype associated with PorB.IA.

The outer membrane transferrin binding protein TbpA has previously been classified into two groups, based on the presence (D) or absence (N) of a deletion in variable region 3 (VR3) (67). However, our data do not support this classification. While we observe three clusters (two major and one minor) (Fig. 4F), both major groups have alleles with and without deletions in VR3. Instead, sequence differences in VR2, and to a lesser extent in VR1, appear to be the primary drivers of differentiation between the two main TbpA clusters. The possible reason for the differences observed between these reports is the small number of sequences analyzed in the previous study. Pajón et al. had access to only nine TbpA sequences in 1997 (67), while we compared TbpA variation among 638 allele sequences derived from nearly 4,000 isolates.

Finally, lipoprotein TbpB, which is involved in scavenging iron from human transferrin in conjunction with TbpA (68, 69), can be broadly classified into two isotypes based on protein size. Both N. gonorrhoeae and hyperinvasive meningococcal strains have type II TbpB (70). However, sequence analyses with 48 *Neisseria* isolates, including both pathogenic and commensal species, indicated that the two TbpB isotypes could be further divided into five families, from A to E. N. gonorrhoeae isolates are found in the TbpB_C_, along with N. meningitidis and commensal species. TbpB_E_ is composed exclusively of N. gonorrhoeae TbpB (71). Consistent with these results, phenotypic analysis clustered N. gonorrhoeae TbpB alleles into two groups (Fig. 4G). The two clusters are not as evolutionarily divergent as other groups observed in this study, likely because they are both members of the type II family. TbpB_E_ (Fig. 4G, green-shaded area) accounts for the majority of alleles (247/320 alleles) and 80% of isolates.

These phylogenetic analyses illustrate the extent of sequence variation among the global N. gonorrhoeae population and highlight antigens’ multiple variants, which may need to be considered in a vaccine design to account for distantly related protein families.

### AniA and FetB are multi-*Neisseria* vaccine candidates based on phylogenetic analyses.

**FIG 5:**
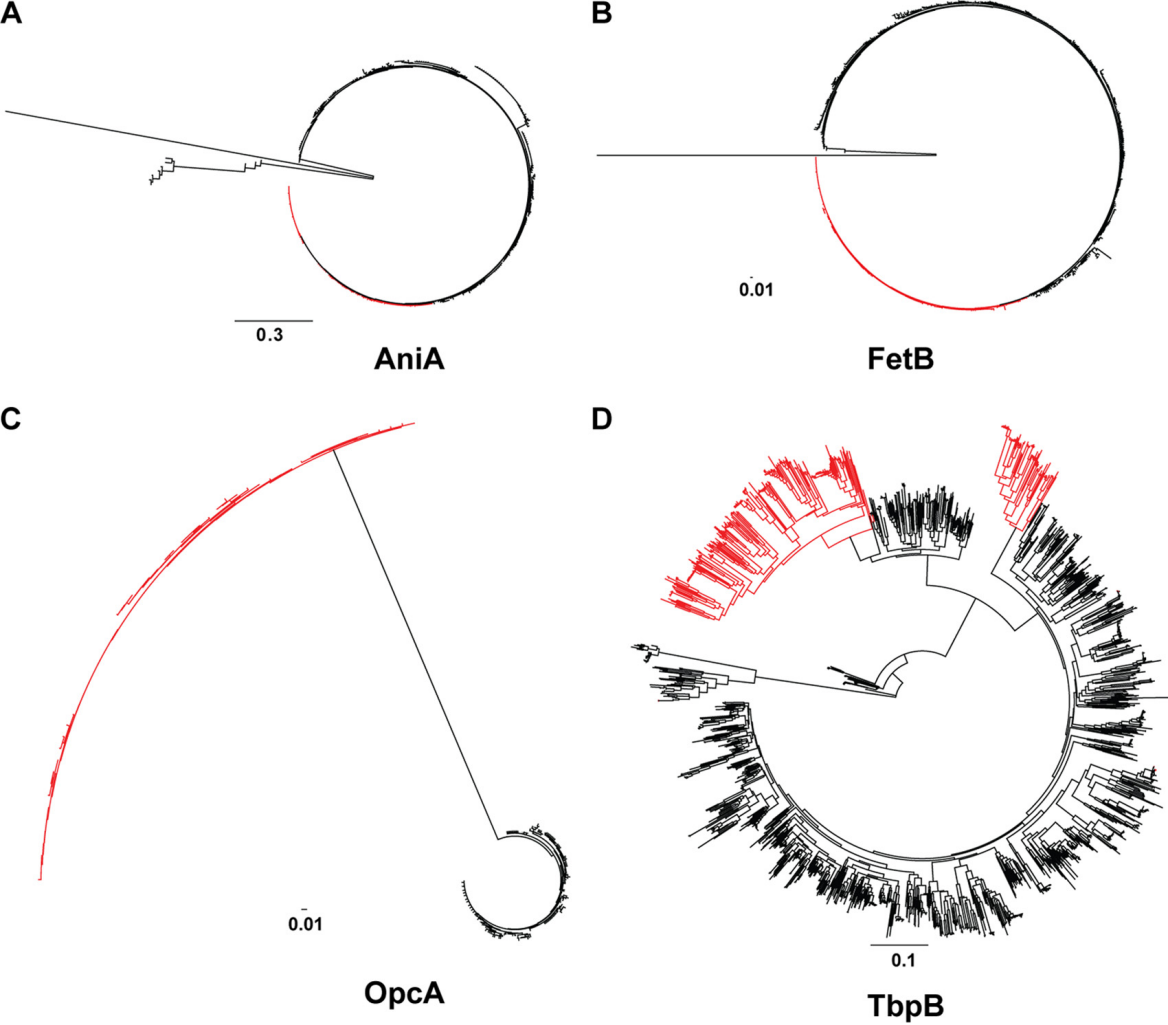
Phylogenetic relationships of AniaA, FetB, OpcA, and TbpB between N. gonorrhoeae and N. meningitidis. Neighbor-joining phylogenetic trees of indicated antigens from N. gonorrhoeae and N. meningitidis were constructed in Geneious using the Jukes-Cantor genetic distance model. N. gonorrhoeae alleles are designated using red branches. (A) AniaA; (B) FetB; (C) OpcA; (D) TbpB.

We subsequently used phylogenetic comparisons to assess whether any gonorrhea vaccine candidate antigens could potentially be considered in a multi-*Neisseria* vaccine to protect against gonorrhea and meningococcal sepsis and meningitis simultaneously. While NGO1559 was highly conserved in N. gonorrhoeae (Fig. 2), it had no N. meningitidis homolog. With rare exceptions, N. gonorrhoeae protein variants clustered separately from their meningococcal homologs (Fig. 5; Fig. S4 to S8). Occasional instances of possible horizontal gene transfer between the species, where a gonococcal variant bundled with an otherwise exclusively N. meningitidis group or vice versa, could be observed for several antigens, including LptD, OmpU, Slam2, TamA, and TbpA (Fig. S4 to S8).

10.1128/mSphere.00977-20.5 FIG S4 Phylogenetic relationships of vaccine candidates (organized in alphabetical order from A to I) between N. gonorrhoeae and N. meningitidis. Neighbor-joining phylogenetic trees of indicated antigens from N. gonorrhoeae and N. meningitidis and not specifically mentioned in the text were constructed in Geneious using the Jukes-Cantor genetic distance model. N. gonorrhoeae alleles are designated using red branches. Download FIG S4, PDF file, 0.3 MB. Copyright © 2021 Baarda et al. 2021 Baarda et al. This content is distributed under the terms of the Creative Commons Attribution 4.0 International license.

Based on phylogenetic comparisons between N. gonorrhoeae and N. meningitidis, the nitrite reductase AniA (Fig. 5A) and the component of the ferric enterobactin transport system FetB (Fig. 5B) seem the most useful candidates for inclusion in a multi-*Neisseria* vaccine. For both proteins, N. gonorrhoeae variants are in the same cluster as the N. meningitidis homologs or are in a closely related grouping. One consideration, however, is that 34% of N. meningitidis isolates have a frameshift mutation that abolishes AniA expression (72). Our analyses showed the existence of a higher percentage (43.8%) of AniA with premature stop codons in meningococcal isolates globally. Similarly, 34 alleles associated with 4,147 N. meningitidis isolates (28.8% of the population) encoded FetB sequences with premature stop codons.

The antigen subfamily separations seen within N. gonorrhoeae sequences (as discussed in a previous section) were further accentuated between N. gonorrhoeae and N. meningitidis isolates (Fig. 5C and D; Fig. S4 to S8). It was especially apparent for OpcA and TbpB. The gonococcal OpcA cluster was evolutionarily distinct from the N. meningitidis group (Fig. 5C). OpcA, an adhesin, was proposed as part of a DNA island imported into N. gonorrhoeae and N. meningitidis genomes from different species, based on the low homology between N. gonorrhoeae and N. meningitidis and the presence of a DNA uptake sequence upstream of the OpcA locus (73). From this standpoint, it appears as the least suitable candidate for a multi-*Neisseria* vaccine. For TbpB, N. gonorrhoeae sequences formed two distinct groups completely separated from each other (Fig. 5D). The larger N. gonorrhoeae cluster corresponded to TbpB_E_ and did not have any N. meningitidis sequences associated with it (Fig. 5D). We noted that the majority of N. gonorrhoeae variants, although clustered separately from the N. meningitidis homologs, were moderately closely related to their meningococcal counterparts.

Together, these investigations suggest that while both AniA and FetB may be attractive candidates for a multi-*Neisseria* vaccine, additional, potentially species-specific antigens will need to be included to compensate for the lack of these proteins in substantial proportions of the N. meningitidis population.

### Polymorphism mapping reveals conserved regions in vaccine antigens.

**FIG 6:**
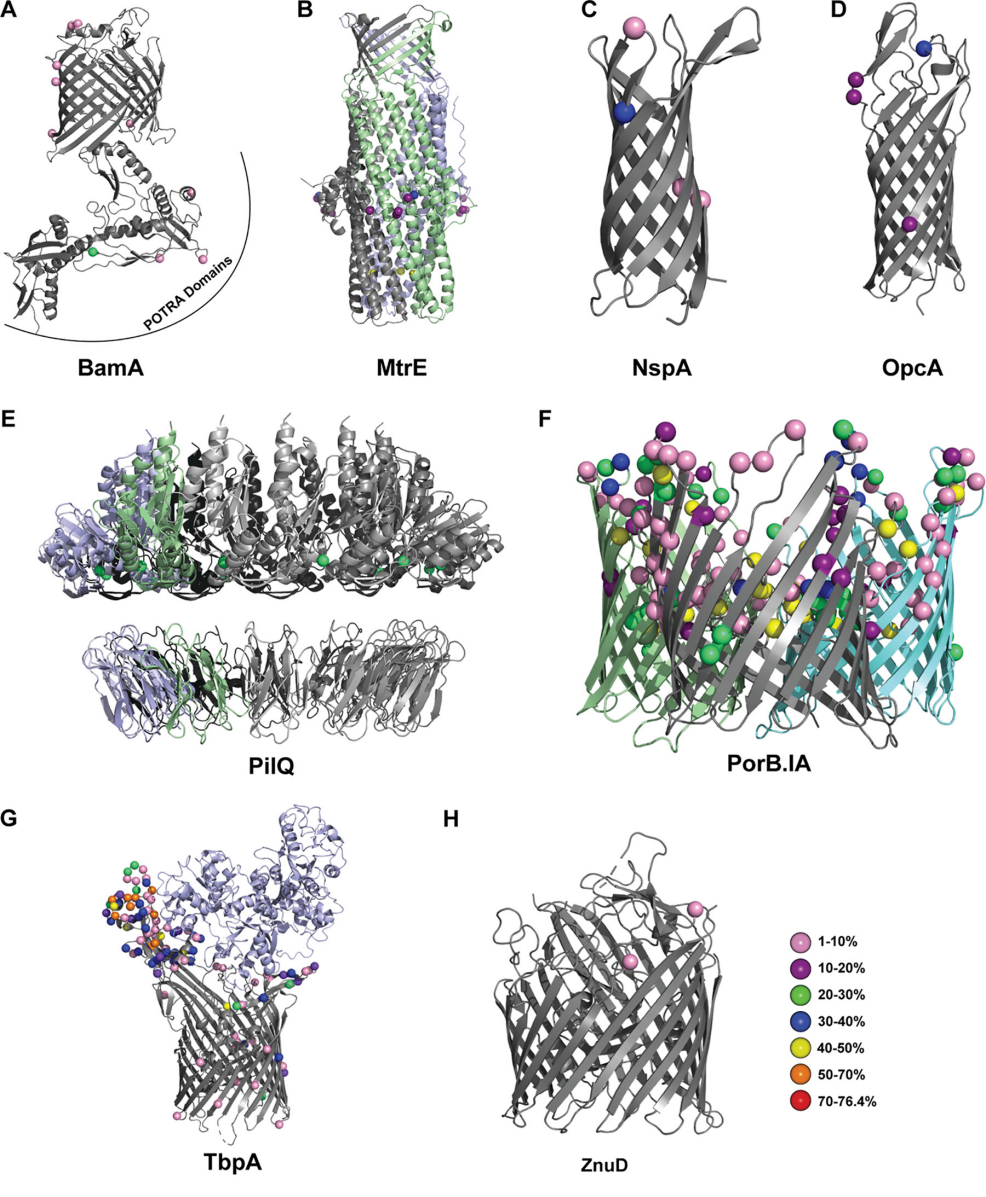
Polymorphism mapping to β-barrel outer membrane protein vaccine candidates. Polymorphic amino acid sites, weighted by the proportion of isolates associated with each polymorphism, were mapped to the structures of BamA (PDB accession no. 4K3B) (A), MtrE (4MT0) (B), NspA (1P4T) (C), OpcA (1K24) (D), PilQ (4AV2) (E), PorB.IA (4AUI) (F), TbpA (3V8X) (G), and ZnuD (4RVW) (H). When applicable, the biologically relevant multimers are shown, with individual monomers in different colors. Human transferrin associated with TbpA is shown in light blue. The polymorphic site color coding used in the figure is as follows: pink, 1 to 10%; purple, 10 to 20%; green, 20 to 30%; blue, 30 to 40%; yellow, 40 to 50%; orange, 50 to 70%; red, 70 to 76.4%.

**FIG 7:**
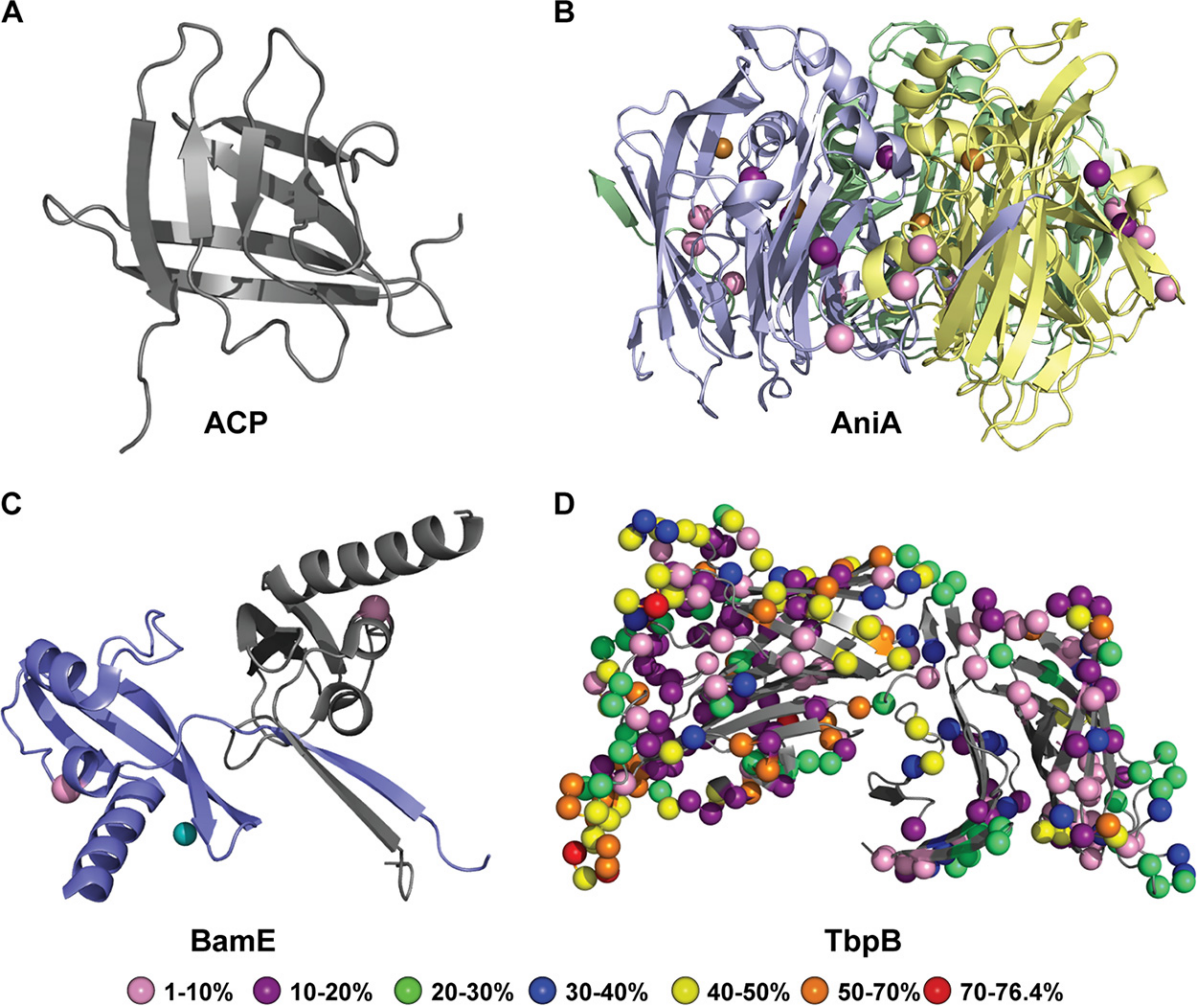
Polymorphism mapping to lipoprotein vaccine candidates. Polymorphic sites, weighted by the proportion of isolates associated with each polymorphism, were mapped to the structures of ACP (6GQ4) (A), AniA (5TB7) (B), BamE (5WAM) (C), and TbpB (3V8U) (D). When applicable, the biologically relevant multimers are shown, with individual monomers in different colors. Copper and zinc ions associated with AniA and BamE are designated by copper-colored and cyan spheres, respectively. Polymorphic site color coding is as follows: pink, 1 to 10%; purple, 10 to 20%; green, 20 to 30%; blue, 30 to 40%; yellow, 40 to 50%; orange, 50 to 70%; red, 70 to 76.4%.

To map antigen polymorphic sites, we used the available eight crystal structures of β-barrelouter membrane proteins (OMPs) (Fig. 6) and four lipoproteins (Fig. 7) from either N. gonorrhoeae or the closely related N. meningitidis. In the past, we have performed similar analyses for the trimeric outer membrane channel component of the multiple transferable resistance (*mtr*) and fatty acid resistance (*far*) efflux pump systems, MtrE (74, 75), and the central component of the β-barrel assembly machinery (BAM) complex, BamA (41). Herein, however, we calculated the prevalence of each polymorphism within the N. gonorrhoeae population. This strategy allows the visual demonstration of the polymorphism frequency, in addition to its location, to determine whether several protein variants should be considered for a broad vaccine coverage (Fig. 6 and 7).

Importantly, only two low-prevalence polymorphisms (1 to 10%) were observed in surface loops of BamA (Fig. 6A). The highest-prevalence polymorphism, found in 20 to 30% of the N. gonorrhoeae population, was present in one of the periplasmic polypeptide transport-associated (POTRA) domains. An additional seven low-prevalence polymorphisms were found throughout the BamA β-barrel and POTRA domains. Remarkably, over 99% of N. gonorrhoeae isolates did not have any surface-exposed polymorphisms in MtrE (Fig. 6B). Per subunit, five polymorphisms (four in 10 to 20% of the population and one in 30 to 40%) are found in the equatorial domain of the periplasmic α-barrel, while a single high-prevalence polymorphism (40 to 50%) is present at the base of the periplasmic channel facing the pore’s interior (Fig. 6B). Similarly, neisserial surface protein A (NspA), which contributes to N. gonorrhoeae serum resistance by binding factor H and factor H-like protein 1 (76), has a single low-prevalence polymorphism (1 to 10% of isolates) in one of its surface loops (Fig. 6C). Three more polymorphisms are present in the β-barrel portion of the protein; two are in 1 to 10% of isolates, and the third is in 30 to 40% (Fig. 6C). The adhesin OpcA exhibits three polymorphisms that are present in 10 to 20% of the population, two in a surface loop and one in the β-barrel portion, and one surface loop polymorphism that is present in 30 to 40% of the population (Fig. 6D). The dodecameric OMP antigen PilQ (77, 78) exhibited a single polymorphic site per subunit, within the structural data available for the periplasmic domains of N. meningitidis PilQ (78), that diverged in 20 to 30% of isolates (Fig. 6E). Further, no three-dimensional data are available for PorB.IB, despite its being the more prevalent subtype. However, mapping PorB.IA variants to the structure revealed extensive differences in all surface-exposed loops and, to a smaller degree, within the β-barrel itself (Fig. 6F). TbpA was highly variable, especially in a series of extracellular loops that extend ∼60 Å above the cell surface (Fig. 6G) (79). This portion included eight sites that diverged from the most common sequence in 50 to 70% of isolates, along with five of the six sites that are polymorphic in 40 to 50% of the population. However, with the exception of D722 (polymorphic in 1 to 10% of isolates), the residues responsible for binding human transferrin were conserved in >99% of the N. gonorrhoeae population (80). In contrast, the zinc uptake protein ZnuD (TdfJ) was highly conserved and had only two surface loop polymorphisms found in 1 to 10% of the population within regions of the protein with available structural data (Fig. 6H).

Finally, ACP showed remarkable conservation of the four lipoprotein vaccine candidates with existing structural information (Fig. 7). The three-dimensional structure of N. gonorrhoeae ACP (81) did not incorporate any polymorphisms present in ≥1% of the population (Fig. 7A). Each subunit of the AniA trimer had four low-prevalence polymorphic sites (1 to 10%) and two sites divergent in 10 to 20% of the population (Fig. 7B). Each subunit of the BamE dimer possessed a single low-prevalence site (1 to 10% of the population) (Fig. 7C). TbpB was the most highly polymorphic lipoprotein examined, with 379 polymorphic sites present in ≥1% of the global N. gonorrhoeae population (Fig. 7D). One hypervariable site diverged from the most common sequence in 76.4% of isolates. Including this site, four positions were polymorphic in >70% of the population, and 31 variants were present in 50 to 70% of isolates.

Cumulatively, these analyses showed that with the exception of PorB, TbpA, and TbpB, all of the OMPs and lipoproteins examined showed relatively few polymorphisms, particularly in the surface-exposed loops that are the most important from the vaccine design standpoint. Thus, BamA, MtrE, NspA, OpcA, ZnuD, ACP, AniA, and BamE appear as promising gonorrhea antigens. Several loops within the TbpA pore are highly conserved and could represent attractive targets for a vaccine that employed recombinant protein loops rather than the entire protein. A vaccine comprising the extracellular TbpA epitopes that elicits functional antibody responses targeting the transferrin binding pocket and occluding the pore might inhibit TbpA function. Similarly, thorough consideration and design would be necessary for TbpB. Indeed, a structure-based design to develop chimeric antigens to circumvent pathogen diversity has been successful in experimental vaccines against both bacterial and viral pathogens (82–85).

### Conclusions.

This is the first large-scale assessment of gonorrhea vaccine antigen variability. Evaluation of allele distributions revealed that >50% of N. gonorrhoeae isolates worldwide had three or fewer alleles for 23 antigens (Fig. 2). Among those, eight were exceptionally well conserved, with a single allele accounting for >80% of global isolates. Generation of phylogenetic trees indicated that the majority of antigens had closely related alleles and also revealed distinct subfamilies for IgA1 protease, MafA, PorB, and TbpB, consistent with classifications established in the literature (Fig. 3). Importantly, mapping polymorphic sites to structural data for 12 antigens showed that the majority of surface-exposed regions were identical in >90% of the global N. gonorrhoeae population (Fig. 6 and 7). Notable exceptions were PorB.IA, TbpA, and TbpB, which were highly polymorphic. However, our analysis revealed conserved surface loops of TbpA, associated with transferrin binding, that could be presented to the immune system separately from the rest of the protein by use of a delivery system, for instance. Considering the high degree of conservation, the distribution among N. gonorrhoeae strains globally, or the low-frequency sequence polymorphisms in surface loops suggests that ACP, AniA, BamA, BamE, MtrE, NspA, NGO0778, NGO1251, NGO1985, OpcA, PldA, Slam2, and ZnuD are promising candidates for a gonorrhea vaccine. Together, these results can inform gonorrhea vaccine development, including structural vaccinology efforts, by identifying conserved antigens, highlighting regions of conservation, and mapping polymorphic sites. Change in an antigen epitope landscape, however, is one of the hindrances in design of a successful vaccine. Important additional factors include bacterial escape from immune surveillance by reducing an antigen’s prevalence/expression on the cell surface and intrinsic and extrinsic host-related, environmental, behavioral, nutritional, and vaccine administration factors (1, 86, 87).

## MATERIALS AND METHODS

### Allele mining, SNP/SAAP, and phylogenetic analyses.

Nucleotide or amino acid sequences for all antigens were downloaded from the *Neisseria* PubMLST database as previously described (41), between January and May 2020. Alleles annotated as atypical or with premature stop codons were excluded from analysis. Nucleotide sequences were aligned in Geneious Prime 2020.1.2 using the Clustal Omega algorithm. The FA1090 allele was set as the reference sequence, automated SNP/SAAP annotation was performed in Geneious, and the results were exported to Excel. The FA1090 LbpA allele possesses a premature stop codon, so the most common allele (16) was used as the reference sequence for this locus (see Data Set S1 in the supplemental material). For phylogenetic and polymorphism mapping analyses, nucleotide sequences were translated and aligned by Muscle in Geneious. Neighbor-joining trees were assembled from aligned amino acid sequence data for each antigen by using the Jukes-Cantor distance model (88) in Geneious.

### Allele proportion analysis.

Using data from the two-field breakdown table from PubMLST, the allele number and the number of isolates associated with each allele were imported into GraphPad Prism v8.4.3 for Mac. “Parts of whole” graphs were generated automatically.

### Polymorphism mapping.

The applicable crystal structure sequence was aligned against translated sequences from all antigen alleles using Muscle in Geneious. The crystal structures of the following proteins were used in this analysis: ACP (PDB accession no. 6GQ4) (81), AniA (5TB7) (89), BamA (4K3B) (90), BamE (5WAM) (91), MtrE (4MT0) (92), NspA (1P4T) (93), OpcA (1K24) (94), PilQ (4AV2) (78), PorB.IA (4AUI) (95), TbpA (3V8X) (80), TbpB (3V8U) (80), and ZnuD (4RVW) (96).

The number of isolates associated with each polymorphic site in the alignment was calculated and divided by the total number of isolates with data for each antigen to determine the proportion of the population that possessed each polymorphism (number of isolates with polymorphism/total number of N. gonorrhoeae isolates). The polymorphisms associated with >1% of isolates were mapped to structural data using PyMOL (https://pymol.org), as previously described (41).

10.1128/mSphere.00977-20.6 FIG S5 Phylogenetic relationships of vaccine candidates (arranged in alphabetical order from L to Ng) between N. gonorrhoeae and N. meningitidis. Neighbor-joining phylogenetic trees of indicated antigens from N. gonorrhoeae and N. meningitidis and not specifically mentioned in the text were constructed in Geneious using the Jukes-Cantor genetic distance model. N. gonorrhoeae alleles are designated using red branches. Download FIG S5, PDF file, 0.3 MB. Copyright © 2021 Baarda et al. 2021 Baarda et al. This content is distributed under the terms of the Creative Commons Attribution 4.0 International license.

10.1128/mSphere.00977-20.7 FIG S6 Phylogenetic relationships of vaccine candidates (organized in alphabetical order from NG to Ns) between N. gonorrhoeae and N. meningitidis. Neighbor-joining phylogenetic trees of indicated antigens from N. gonorrhoeae and N. meningitidis and not specifically mentioned in the text were constructed in Geneious using the Jukes-Cantor genetic distance model. N. gonorrhoeae alleles are designated using red branches. Download FIG S6, PDF file, 0.4 MB. Copyright © 2021 Baarda et al. 2021 Baarda et al. This content is distributed under the terms of the Creative Commons Attribution 4.0 International license.

10.1128/mSphere.00977-20.8 FIG S7 Phylogenetic relationships of vaccine candidates (organized in alphabetical order from O to P) between N. gonorrhoeae and N. meningitidis. Neighbor-joining phylogenetic trees of indicated antigens from N. gonorrhoeae and N. meningitidis and not specifically mentioned in the text were constructed in Geneious using the Jukes-Cantor genetic distance model. N. gonorrhoeae alleles are designated using red branches. Download FIG S7, PDF file, 0.7 MB. Copyright © 2021 Baarda et al. 2021 Baarda et al. This content is distributed under the terms of the Creative Commons Attribution 4.0 International license.

10.1128/mSphere.00977-20.9 FIG S8 Phylogenetic relationships of vaccine candidates (presented in alphabetical order from S to Z) between N. gonorrhoeae and N. meningitidis. Neighbor-joining phylogenetic trees of indicated antigens from N. gonorrhoeae and N. meningitidis and not specifically mentioned in the text were constructed in Geneious using the Jukes-Cantor genetic distance model. N. gonorrhoeae alleles are designated using red branches. Download FIG S8, PDF file, 0.5 MB. Copyright © 2021 Baarda et al. 2021 Baarda et al. This content is distributed under the terms of the Creative Commons Attribution 4.0 International license.
