# Supplementary material for: Comprehensive Bioinformatic Assessments of the Variability of Neisseria gonorrhoeae Vaccine Candidates
Source: mSphere. 2021 Feb 3;6(1):e00977-20. doi: 10.1128/mSphere.00977-20 (PMC7860988; doi:10.1128/mSphere.00977-20)
Supplement: TABLE S1 [file mSphere.00977-20-st001.pdf]

Supplemental Table S1. Antigens with altered alleles. Alleles containing internal stop codon were eliminated from further bioinformatic analyses.

Antigen	Internal stop codon	Atypical	Frameshift	No start codon
**ACP**	0	0	0	0
**AniA**	0	0	0	0
**BamA**	0	0	0	0
**BamE**	0	0	0	0
**NGO1985**	0	0	0	0
**CsgG (NGO0834)**	6	0	0	1
**FetB (NGO2092)**	0	0	0	0
**IgA1 protease**	0	0	0	0
**IgA2 protease**	16	0	16	0
**LbpA**	31	0	0	0
**LolB (NGO0439)**	0	0	0	0
**LprI**	0	0	0	0
**LptD**	0	1	0	0
**MafA (NGO1067)**	2	0	0	0
**MetQ**	0	0	0	0
**MtrE**	4	0	3	0
**NgMIP (NGO1225)**	0	0	0	0
**NGO0425**	0	0	0	0
**NGO0778**	0	0	0	0
**NGO1251**	0	0	0	0
**NGO1344**	1	0	0	0
**NGO1559**	0	0	0	0
**NspA (NGO0233)**	2	1	1	0
**OmpU (NGO1688)**	0	0	0	0
**OpcA (NGO0868)**	4	0	0	0
**PilN (NGO0097)**	0	0	0	0
**PilQ**	18	1	0	0
**PldA**	0	0	0	0
**PorB**	0	0	0	0
**Slam2**	0	0	0	0
**SliC**	0	0	0	0
**TamA**	0	0	0	0
**TbpA**	1	0	0	0
**TbpB**	3	0	0	0
**ZnuD, TdfJ (NGO1205)**	1	0	0	0
