# Supplementary figures and images for: Comprehensive Bioinformatic Assessments of the Variability of Neisseria gonorrhoeae Vaccine Candidates
Source: mSphere. 2021 Feb 3;6(1):e00977-20. doi: 10.1128/mSphere.00977-20 (PMC7860988; doi:10.1128/mSphere.00977-20)

Supplemental Figure S1

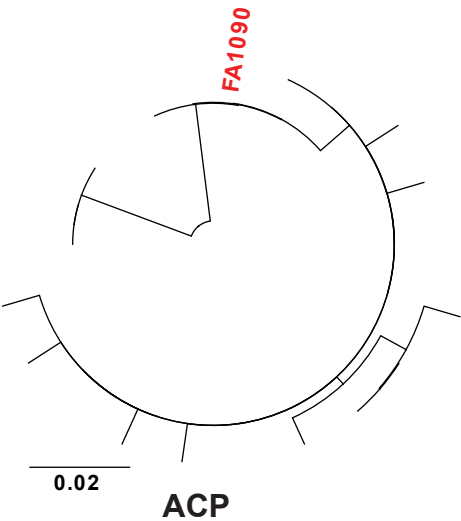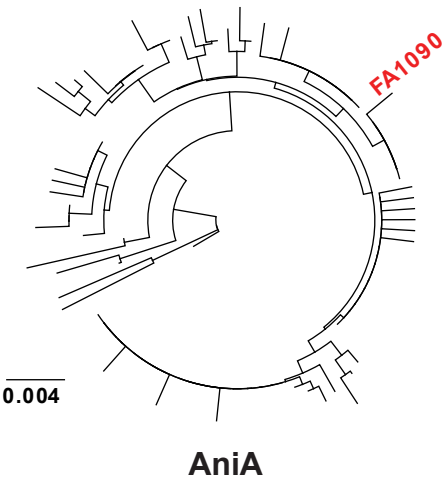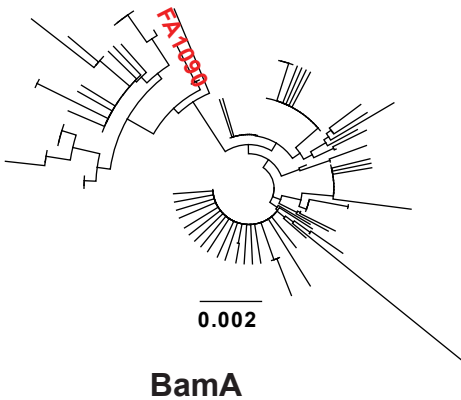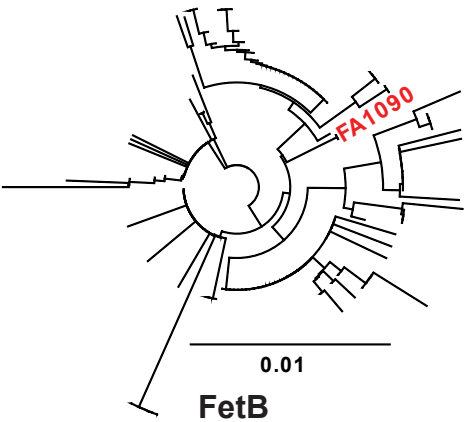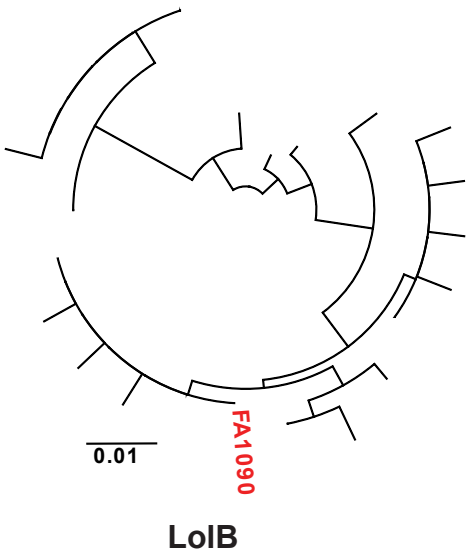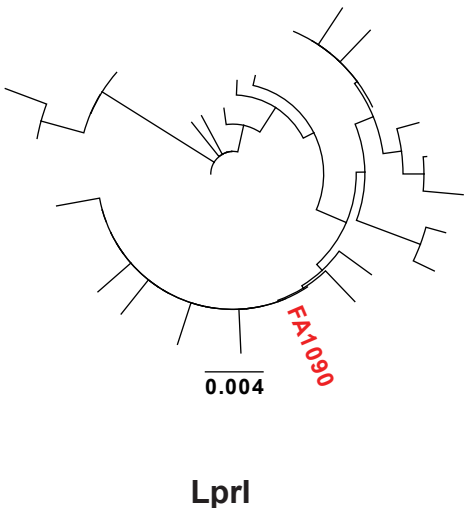

Supplement: FIG S1 [file mSphere.00977-20-sf001.pdf]

Supplemental Figure S2

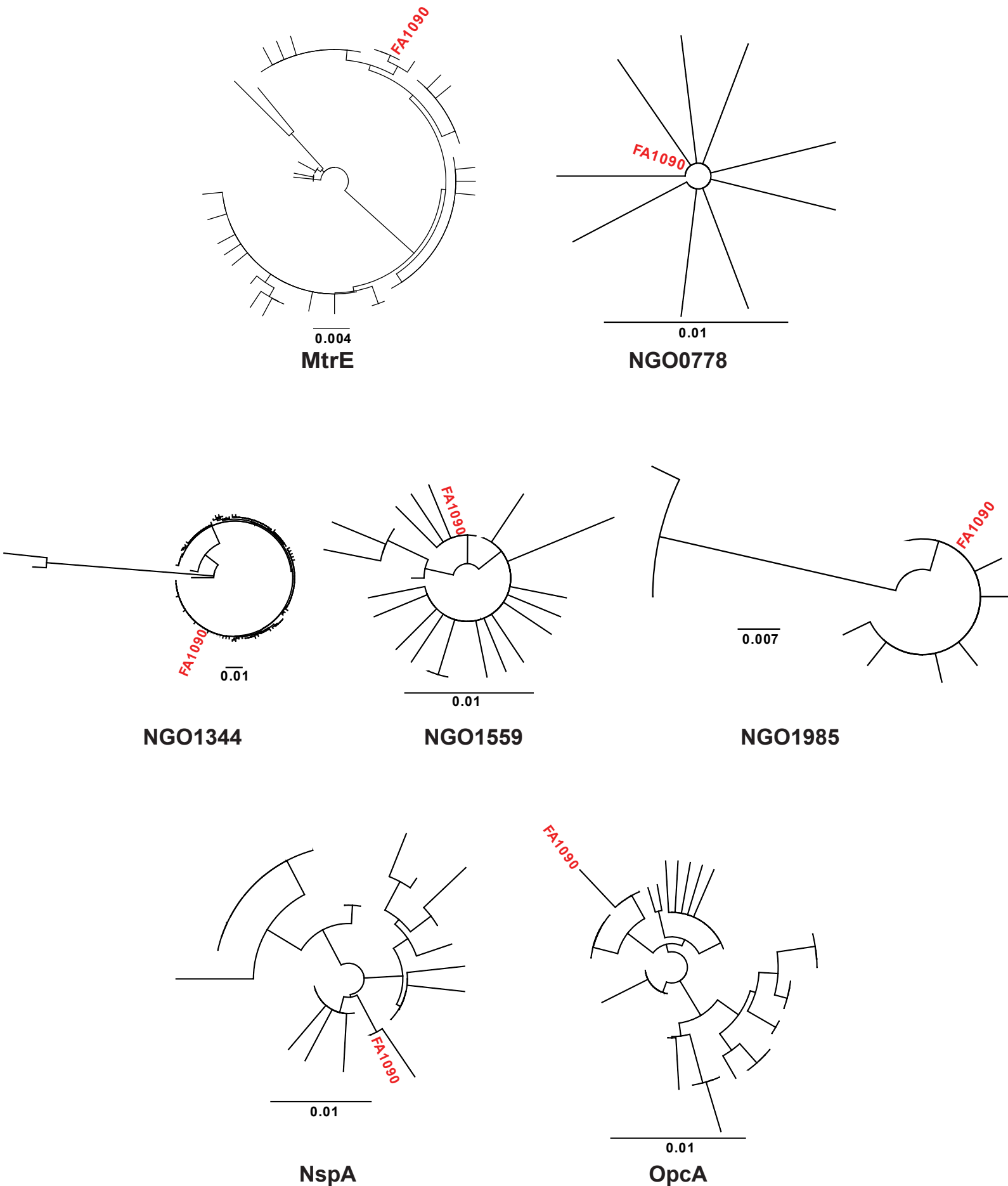

Supplement: FIG S2 [file mSphere.00977-20-sf002.pdf]

Supplemental Figure S3

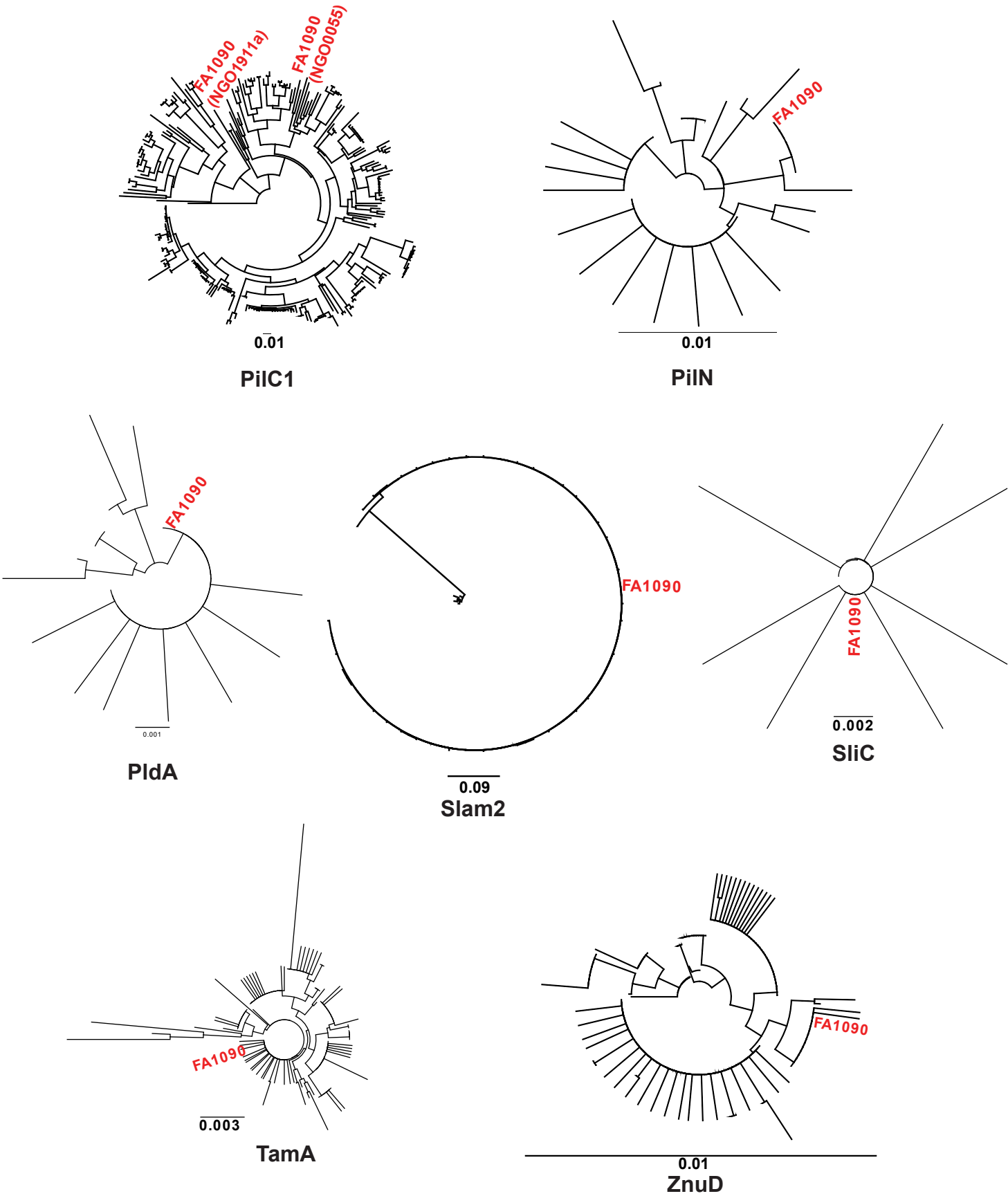

Supplement: FIG S3 [file mSphere.00977-20-sf003.pdf]

Supplemental Figure S4

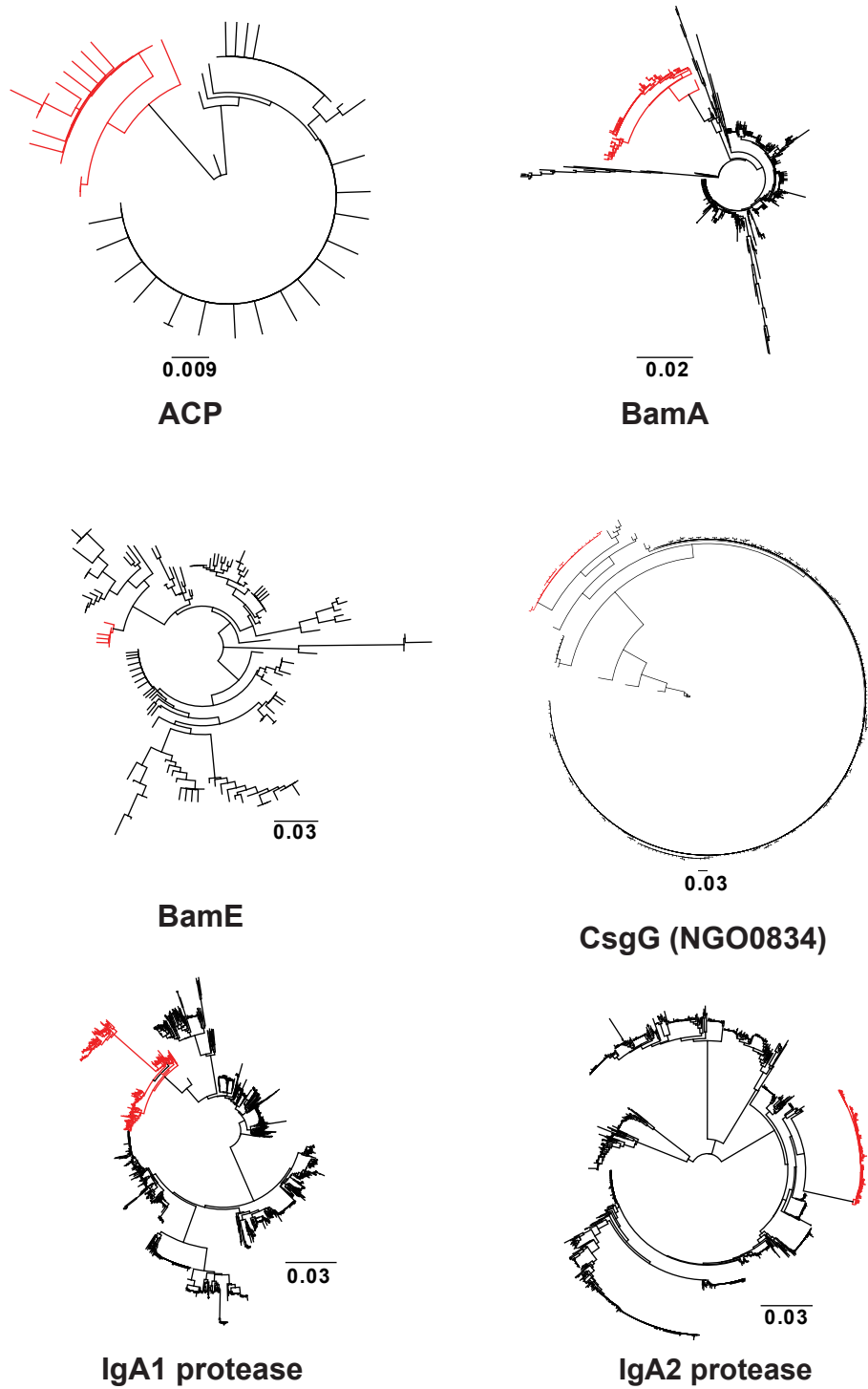

Supplement: FIG S4 [file mSphere.00977-20-sf004.pdf]

Supplemental Figure S5

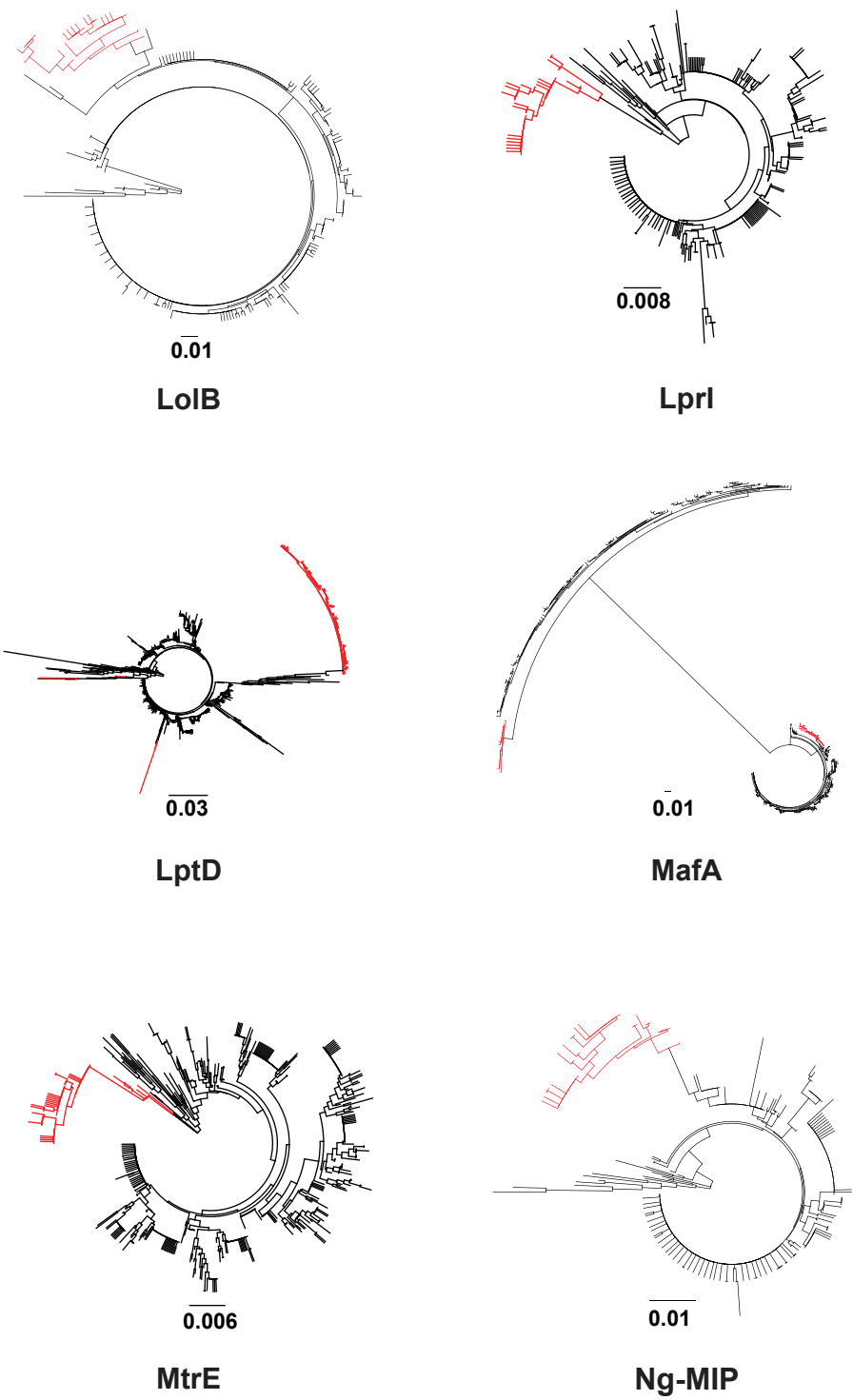

Supplement: FIG S5 [file mSphere.00977-20-sf005.pdf]

Supplemental Figure S6

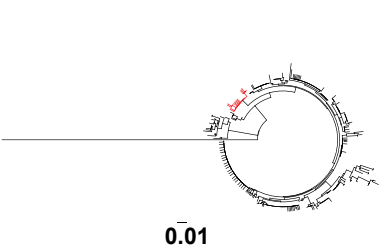

NGO0425

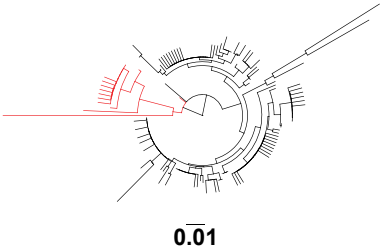

NGO0778

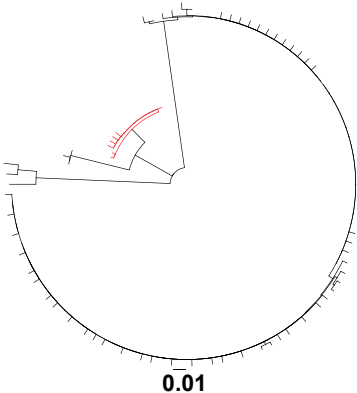

NGO1251

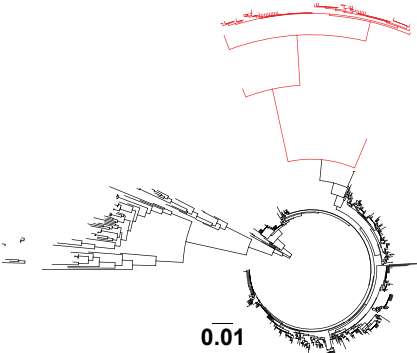

NGO1344

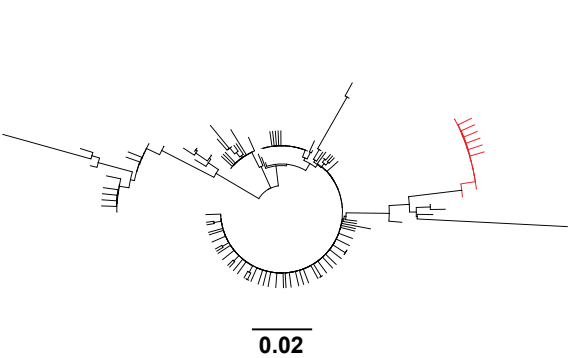

NGO1985

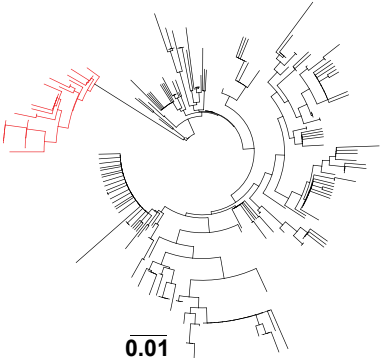

NspA

Supplement: FIG S6 [file mSphere.00977-20-sf006.pdf]

Supplemental Figure S7

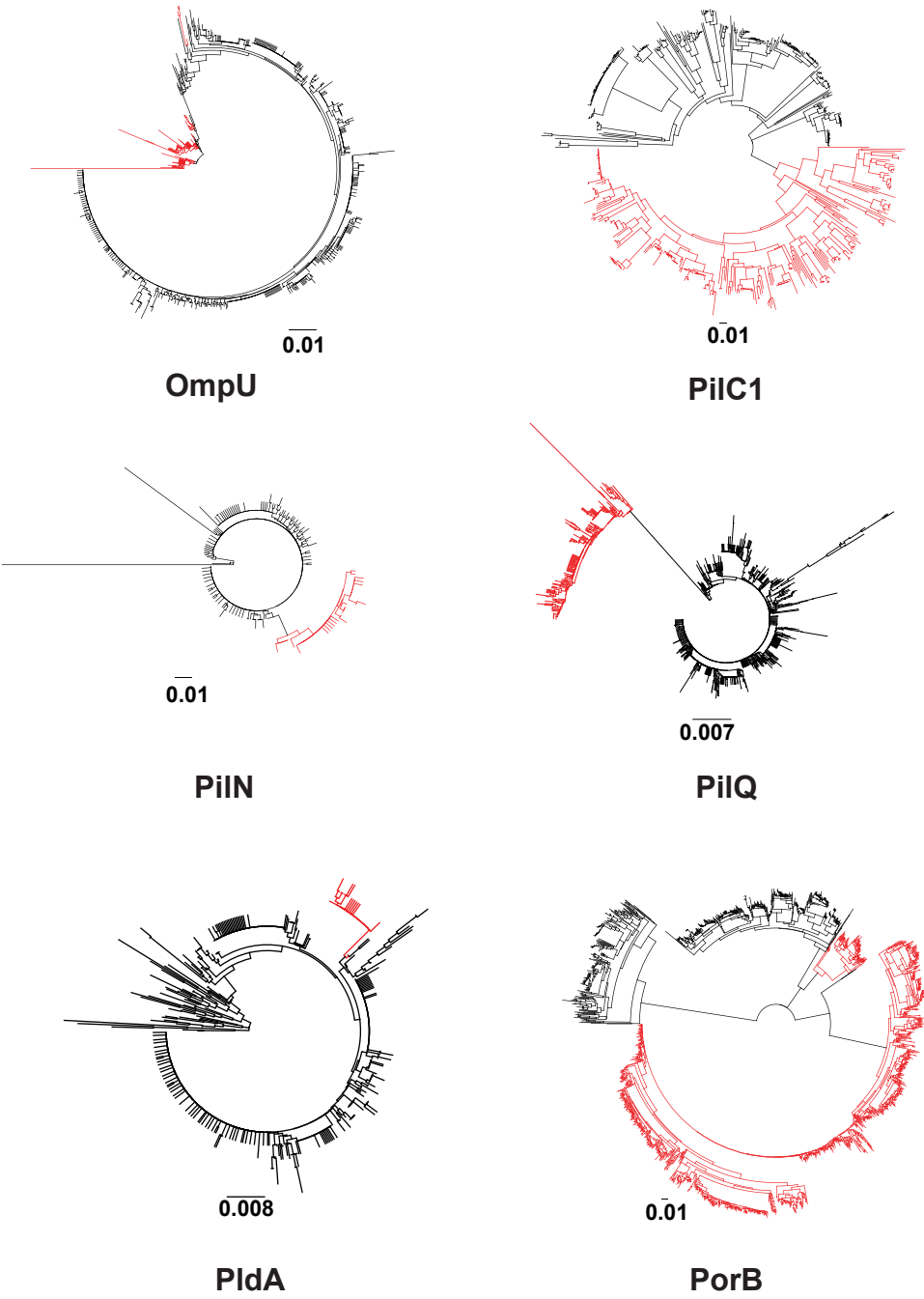

Supplement: FIG S7 [file mSphere.00977-20-sf007.pdf]

Supplemental Figure S8

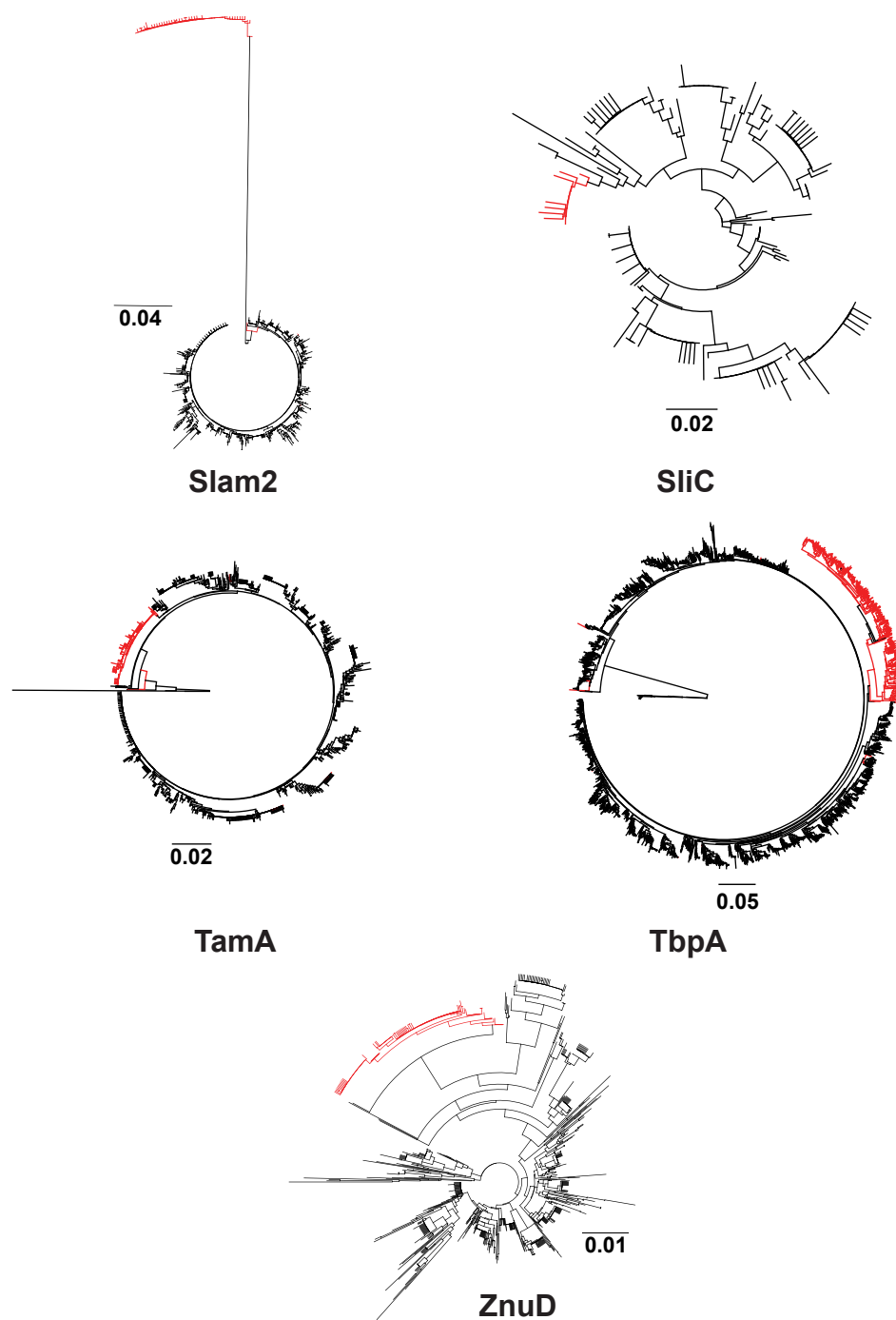

Supplement: FIG S8 [file mSphere.00977-20-sf008.pdf]
